# Dose-Dependent Acute Circulatory Fates Elicited by Cadmium Are Mediated by Differential Engagements of Cardiovascular Regulatory Mechanisms in Brain

**DOI:** 10.3389/fphys.2019.00772

**Published:** 2019-06-18

**Authors:** Shu-Mi Chen, Suttinee Phuagkhaopong, Chi Fang, Jacqueline C. C. Wu, Ya-Hui Huang, Pornpun Vivithanaporn, Hsun-Hsun Lin, Ching-Yi Tsai

**Affiliations:** ^1^Master and Ph.D. Program in Pharmacology and Toxicology, School of Medicine, Tzu Chi University, Hualien City, Taiwan; ^2^Department of Pharmacy, Lotung Poh-Ai Hospital, Yilan City, Taiwan; ^3^Kaohsiung Chang Gung Memorial Hospital, Institute for Translational Research in Biomedicine, Kaohsiung, Taiwan; ^4^Department of Pharmacology, Faculty of Science, Mahidol University, Bangkok, Thailand; ^5^Department of Physiology School of Medicine, Tzu Chi University, Hualien City, Taiwan

**Keywords:** cadmium, bioenergetic failure, anoxia, rostral ventrolateral medulla, cerebral autoregulation, mitochondrial membrane potential

## Abstract

Whereas cadmium is a toxicant that has been shown to cause cardiovascular toxicity and mortality in mammals, few mechanistic studies address its acute circulatory actions. The present study assessed the hypothesis that cadmium effects dose-dependent acute circulatory fates via differential participation of the cardiovascular regulatory mechanisms in brain. In Sprague-Dawley rats maintained under propofol anesthesia, cadmium acetate (8 mg/kg, iv) induced significantly high mortality rate within 10 min, concomitant with progressive decline toward zero level of mean arterial pressure (MAP), heart rate (HR), baroreflex-mediated sympathetic vasomotor tone, and carotid blood flow (CBF). There were concurrent tissue anoxia, cessation of microvascular perfusion, reduction of mitochondrial membrane potential and ATP production, and necrotic cell death in the rostral ventrolateral medulla (RVLM), the brain stem site that maintains blood pressure and sympathetic vasomotor tone. On the other hand, a lower-dose of cadmium (4 mg/kg, iv) resulted in only a transient decrease in MAP that was mirrored by an increase in CBF and baroreflex-mediated sympathetic vasomotor tone, minor changes in HR, along with transient hypoxia, and apoptotic cell death in RVLM. We conclude that cadmium elicits dose-dependent acute cardiovascular effects with differential underlying biochemical and neural mechanisms. At a higher-dose, cadmium induces high mortality by effecting acute cardiovascular collapse via anoxia, diminished tissue perfusion, mitochondrial dysfunction and bioenergetics failure that echo failure of cerebral autoregulation, leading to necrosis, and loss of functionality in RVLM. On the other hand, a lower-dose of cadmium elicits low mortality, transient decrease in arterial pressure, and hypoxia and apoptosis in RVLM that reflect sustained cerebral autoregulation.

## Introduction

Cadmium is a heavy metal and an environmental toxicant. Acute cadmium exposure induces cardiovascular toxicity that may lead to mortality (Dote et al., 2007; Miura et al., 2012). A majority of the mechanistic reports on cardiovascular toxicity elicited by cadmium is based on either long-term exposure to this toxicant (Puri, 1999; Yoopan et al., 2008; Ozturk et al., 2009) or on exposure to cadmium *in vitro* (Majumder et al., 2008; Messner and Bernhard, 2010; Messner et al., 2016) or *ex vivo* (Kisling et al., 1993; Wakabayashi et al., 1995) studies. Much fewer studies address the acute circulatory fates of cadmium, particularly in term of the cardiovascular regulatory mechanisms in brain.

By passing the blood-brain barrier (BBB) and accumulating in the central nervous system (CNS), cadmium may induce neurotoxicity (Wang and Du, 2013) by acting directly on the central circulatory regulatory mechanisms. One potential target of cadmium is the rostral ventrolateral medulla (RVLM), a key neural substrate in the baroreflex neural circuit that is intimately involved in the maintenance of stable blood pressure and sympathetic vasomotor tone (Dampney, 1994; Spyer, 1994). Chang et al. (2009) demonstrated that bioenergetic failure, leading to necrotic cell death, accounts for the loss of functional integrity in RVLM. Clinical studies (Kuo et al., 1997b; Yien et al., 1997; Yen et al., 2000) demonstrated that the resultant defunct baroreflex-mediated sympathetic vasomotor tone is causally related to brain stem death in comatose patients.

The brain uses ∼20% of total body’s oxygen for normal function, making tight regulation of blood flow and oxygen delivery to the brain critical for survival (Widmaier et al., 2006; Bélanger et al., 2011). As such, cerebral autoregulation, which plays an important role in the maintenance of constant blood flow to brain, is another potential target for cadmium-induced neurotoxicity. Of particular interest is that the degree of tissue hypoxia in RVLM is a crucial determinant of the severity of brain stem circulatory regulatory dysfunction (Chang et al., 2009; Chan et al., 2011; Li et al., 2012), which is dependent on whether necrosis or apoptosis has ensued (Chang et al., 2009; Li et al., 2012).

The present study assessed the hypothesis that cadmium mediates dose-dependent acute circulatory fates via differential participation of the cardiovascular regulatory mechanisms in brain. Our physiological and biochemical results showed that a lower-dose of cadmium elicited low mortality, transient decrease in arterial pressure, and hypoxia and apoptosis in RVLM that reflect sustained cerebral autoregulation. On the other hand, a higher-dose of cadmium induced high mortality with a short latency by effecting cardiovascular collapse via anoxia, diminished tissue perfusion, mitochondrial dysfunction and bioenergetics failure that echo failure of cerebral autoregulation, leading to necrosis in RVLM.

## Materials and Methods

### Experimental Animals

All experimental procedures carried out in this study were approved by the Institutional Animal Care and Use Committee of the Kaohsiung Chang Gung Memorial Hospital (approval number: 2017091402), and were in accordance with the guidelines for animal care and use set forth by that committee. Adult male Sprague-Dawley rats (255–322 g; *n* = 159) purchased from BioLASCO, Taiwan, were used. They were housed in an AAALAC International-accredited Center for Laboratory Animals, with maintained room temperature (24 ± 1°C) and 12 h:12 h light/dark cycle (light on at 06:00). Standard laboratory rat chow and tap water were available *ad libitum*.

### Recording of Hemodynamic Parameters

As in our previous studies (Tsai et al., 2016, 2017b), animals were initially anesthetized with an induction dose of pentobarbital sodium (50 mg/kg), given intraperitoneally, to carry out tracheotomy for intubation of the trachea, and cannulation of a femoral artery or vein. Maintenance of anesthetic level was provided by intravenous infusion of propofol (Fresenius Kabi, Graz, Austria) at 25 mg/kg/h. Arterial pressure (AP) recorded from the femoral artery was subjected to continuous, on-line and real-time spectral analysis (Notocord, Croissy-sur-Seine, France). We were particularly interested in the low-frequency (BLF; 0.25–0.8 Hz) band, which takes origin from RVLM (Kuo et al., 1997a), and its power density reflects the prevalence of baroreflex-mediated sympathetic vasomotor tone (Li et al., 2001; Poon et al., 2016). Heart rate (HR) was derived from the AP signals. During the recording session, animals were allowed to breathe spontaneously with room air. The head of the animal was fixed to a stereotaxic head holder (Kopf, Tujunga, CA, United States), and the body temperature was maintained at 37°C by a heating pad.

### Intravenous Administration of Cadmium

Cadmium solutions were prepared by dissolving cadmium acetate (Sigma-Aldrich, St. Louis, MO, United States) in 0.9% saline. The pH of cadmium solution was 7.4. To avoid drug accumulation, each animal received a single dose of cadmium (4 or 8 mg/kg) via the femoral vein. These doses were selected from preliminary experiments as representative doses that elicited differential effects on our experimental parameters within 10 min after administration.

### Measurement of Blood Flow

Carotid blood flow (CBF) was recorded by a transit-time blood flowmeter (Transonic, Ithaca, NY, United States). The transonic flow probe (1.0 mm V-series) was placed around a carotid artery for blood flow recording. The volume flow and the real-time pulsatile flow were recorded.

### Measurement of Tissue Oxygen Level, Microvascular Perfusion, and Temperature

A combined oxygen/temperature/blood flow probe designed for simultaneous and continuous measurement of tissue oxygen tension, blood flow and temperature (Oxford Optronix, Abingdon, United Kingdom) was stereotaxically positioned into RVLM (Chang et al., 2009). Instantaneous changes in local oxygen tension, compensated for fluctuations in tissue temperature, were processed by an OxyLite monitor (Oxford Optronix). Real-time microvascular red blood cell perfusion in tissue was processed by an OxyFlo monitor (Oxford Optronix). The laser Doppler signals from the tissue were recorded in blood perfusion units (BPU), which is a relative unit defined against a controlled motility standard.

### Collection of Tissue Samples

At the conclusion of the recording sessions, animals were perfused with warm saline for the collection of medullary tissues (Tsai et al., 2016, 2017b). A slice of medulla oblongata that contains RVLM (0.5–1.5 mm rostral to the obex) was obtained, and tissues from both sides of the ventrolateral medulla were subsequently collected by micropunches made with a 1 mm (i.d.) stainless steel bore to cover the anatomical boundaries of RVLM, and were stored immediately in liquid nitrogen.

### Determination of Apoptotic Cell Death and Activated Caspase-3

Rostral ventrolateral medulla tissue samples were homogenized for the determination of apoptotic cell death using a cell death ELISA kit (Roche, Mannheim, Germany) that measured the level of histone-associated DNA fragments in cytoplasm. A caspase-3 colorimetric assay kit (BioVision, Mountain View, CA, United States) was used to quantify activated caspase-3.

### Measurement of ATP Content and ADP/ATP Ratio

Total ATP level from homogenized RVLM tissue samples was measured by an ATP detection assay kit (Cayman, Ann Arbor, MI, United States) according to the manufacturer’s instructions. Light emitted from a luciferase-mediated reaction and measured by a luminometer (Berthold Centro LB 960, Bad Wildbad, Germany) was used to calculate the measured values.

ADP/ATP ratio was measured by an ADP/ATP ratio assay kit (Abcam, Cambridge, MA, United States) based on bioluminescent detection of ADP and ATP levels; ADP level was measured by its conversion to ATP. According to the manufacturer’s recommendation, we collected fresh RVLM tissue samples and prepared single cell suspensions within 30 min before the assay, and all procedure was conducted at 4°C. The ADP/ATP ratio was determined by [Data D–Data C]/[Data B–Data A]. Data A, background signal of reaction mix; Data B, sample signal ∼2 min after addition of cells to reaction mix (for the ATP generated by the cells when they are incubated with mix); Data C, sample signal prior to addition of ADP converting enzyme to cells (for the total ATP released and present in the sample); Data D, sample signal ∼2 min after addition of ADP converting enzyme to cells (after conversion of ADP from the samples to ATP).

### Histological Examination

As in our previous studies (Su et al., 2016; Tsai et al., 2017a), the brain stem was fixed in 4% paraformaldehyde for 24 h. The tissues were dehydrated, embedded in paraffin, sectioned at a thickness of 4 μm, and stained by hematoxylin and eosin for histopathological examination. The stained brain stem sections were digitally scanned with a slide Scanner (Pannoramic MIDI, 3DHistech, Budapest, Hungary).

### *In situ* Detection of Apoptosis

For *in situ* detection of apoptosis in the brain stem, a Click-iT Plus TUNEL assay with Alex Fluor 488 dyes (Life Technologies, Carlsbad, CA, United States) was used to process free-floating frozen sections of medulla oblongata for double immunofluorescence staining. According to the manufacturer’s instructions, TUNEL assay dye and a mouse monoclonal antiserum (Life Technologies) against a specific neuronal marker, neuron-specific nuclear protein (NeuN), were first added, followed by incubation with a goat anti-mouse I gG conjugated with Alexa Fluor 568 (Life Technologies). Images were viewed with an Olympus Fluoview 1000 (Tokyo, Japan) laser scanning confocal microscope.

### Flow Cytometry

As described previously (Mattiasson et al., 2003; Zhang L. et al., 2016) with modifications, fresh RVLM tissue was washed with ice-cold phosphate-buffered saline and homogenized with Kontes Dounce homogenizer. The homogenate was placed into microcentrifuge tubes to isolate mitochondria by following the instructions of a Mitochondria isolation kit for tissue (Pierce, Rockford, IL, United States). 20 μg of isolated mitochondria were stained with nonyl acridine orange (NAO; 100 nM, Ex/Em: 488 nm/525 nm; Life Technologies) to evaluate mitochondrial mass and 1,1′,3,3,3′,3′-hexamethylindodicarbocyanine iodide [DiIC_1_(5); 10 nM, Ex/Em: 638 nm/658 nm, Life Technologies] to verify mitochondrial membrane potential. Stained mitochondria were detected by Gallios flow cytometer (Beckman Coulter, Indianapolis, IN, United States), and data were analyzed by Kaluza software (Beckman Coulter). To exclude debris, samples were gated based on light-scattering properties in the forward-scattered light (FS) and side-scattered light (SS) modes, and 20,000 events for each sample within the gate were collected.

### Measurement of NF-κB Activation

Activation of the transcription factor NF-κB was measured by a sensitive colorimetric assay (TransAM NF-κB p65; Active Motif; Carlsbad, CA, United States) according to the manufacturer’s protocol. Briefly, nuclear protein extracted from RVLM was incubated with an immobilized oligonucleotide containing the NF-κB consensus-binding site. This was followed by incubation with a primary NF-κB p65 antibody, and a secondary peroxidase-conjugated antibody at room temperature. After a colorimetric reaction, the optical density was read at 450 nm using an ELISA microtiter plate reader.

### Western Bolt Analysis

The concentration of proteins extracted from RVLM was determined by the BCA assay (Pierce). Western blot analysis was carried out on IL-8 (Abcam, Cambridge, MA, United States) and β-actin (Chemicon, Temecula, CA, United States). Specific antibody-antigen complex was detected using an enhanced chemiluminescence western blot detection system. The amount of protein level was expressed as the ratio relative to β-actin protein.

### Blood Analysis

Blood samples were collected after cadmium administration. 0.1 ml of arterial blood was used to determine pH, sodium and potassium by the epoc Blood Analysis System (Siemens, Erlangen, Germany). 0.1 ml of plasma was subjected to detection of thrombomodulin level using an ELISA assay (BlueGene Biotech; Shanghai, China).

### Statistical Analysis

All values are expressed as mean ± SEM. One-way or two-way analysis of variance with repeated measures, as appropriate, were used to assess group means, followed by Fisher Exact test, Tukey test or Scheffé multiple range test for *post hoc* assessment of individual means. *P <* 0.05 were considered statistically significant.

## Results

### Cadmium Acutely and Dose-Dependently Reduces Survival

**FIGURE 1:**
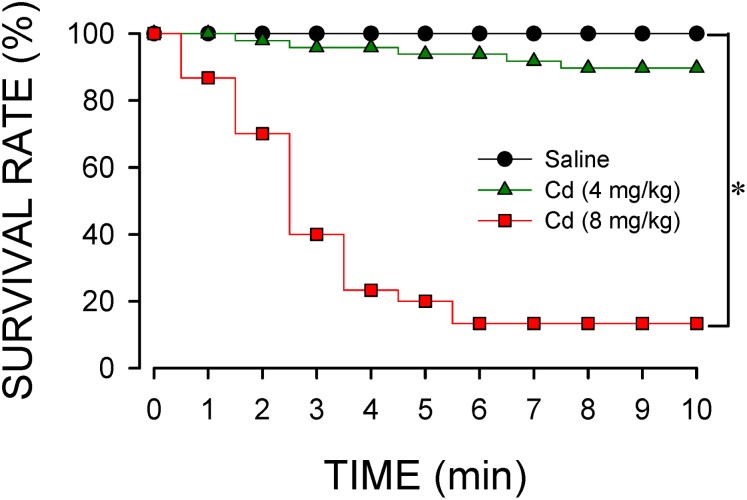
Temporal changes in survival rate in animals that received intravenous administration of cadmium (Cd; 4 or 8 mg/kg) or saline. *n* = 30–48 animals per experimental group. ^*^P < 0.05 vs. saline group in the Fisher Exact test.

Intravenous administration of cadmium elicited a dose-dependent decrease in survival rate (Figure 1). At the lower-dose (4 mg/kg, iv), cadmium induced a progressive reduction in survival rate to 89.6% during the first 10 min of observation. Increasing the dose to 8 mg/kg (iv) further diminished the survival rate to 14.3% by 10 min post-cadmium. Survival rate was not affected by intravenous administration of saline (vehicle control).

### Cadmium Induces Differential and Dose-Related Changes in Arterial Pressure, Heart Rate, or Carotid Blood Flow

**FIGURE 2:**
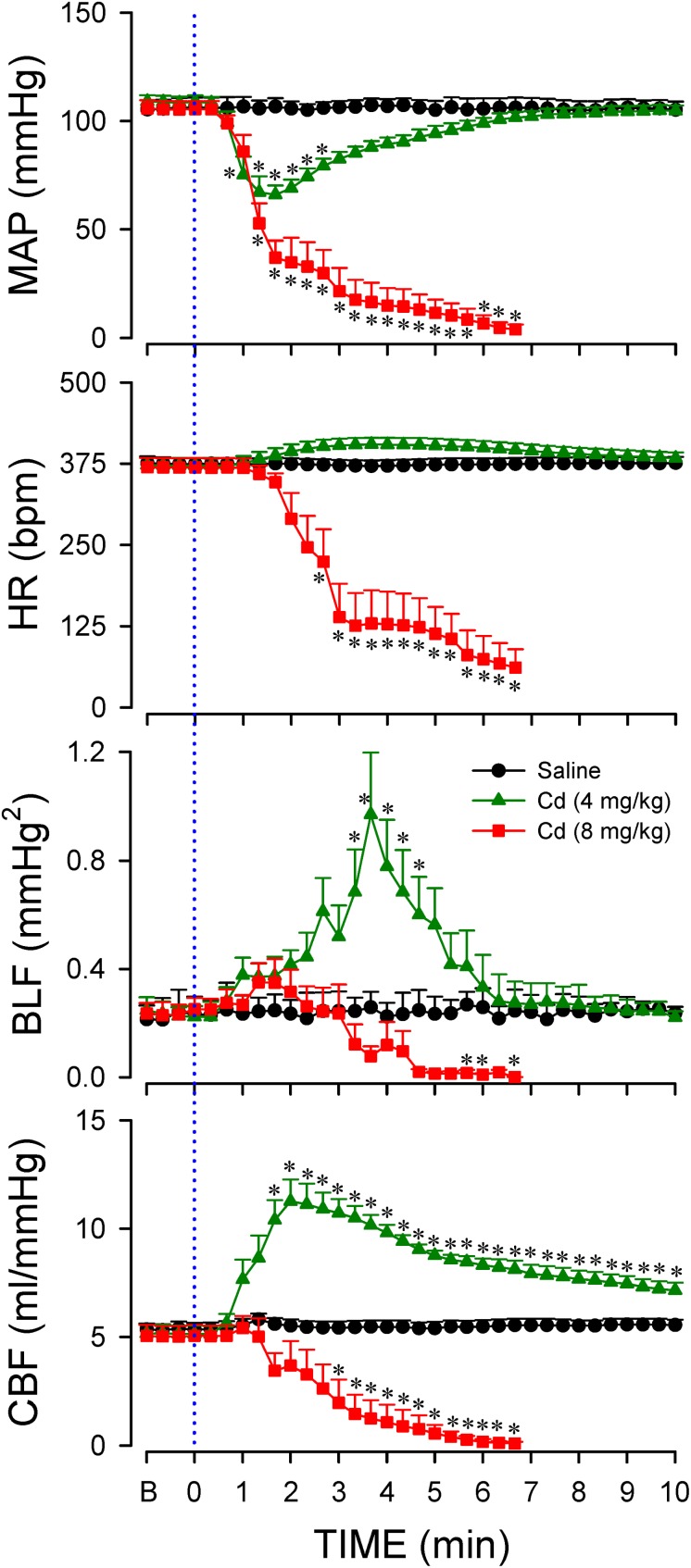
Temporal changes in mean arterial pressure (MAP), heart rate (HR), power density of the low-frequency (BLF) component of arterial pressure spectrum, or carotid blood flow (CBF) in animals that received intravenous administration of cadmium (Cd; 4 or 8 mg/kg) or saline. Values are mean ± SEM, *n* = 6–8 animals per experimental group. ^*^P < 0.05 vs. saline group at corresponding time points in the *post hoc* Scheffé multiple-range test. Blue dotted line denotes time of cadmium injection. B denotes baseline values.

Data shown in Figure 2 demonstrated that intravenous administration of cadmium (4 or 8 mg/kg) elicited differential and dose-related changes in AP, HR, or CBF. Administration of the lower-dose of cadmium (4 mg/kg, iv) elicited a transient hypotension, accompanied by insignificant changes in HR. Concurrently, cadmium significantly augmented the power density of the BLF component of the AP spectrum to reflect the response of the baroreflex-mediated sympathetic vasomotor tone, along with significantly increased CBF. On the other hand, administration of the higher-dose of cadmium (8 mg/kg, iv) resulted in a progressive reduction in mean AP (MAP), HR, and CBF (Figure 2) that paralleled temporally with the decline in survival rate (Figure 1). The BLF power underwent a transient, albeit insignificant increase, followed by a steady decline toward zero level that also paralleled temporally with survival rate. The cardiovascular parameters, however, were not affected by intravenous administration of saline (vehicle control).

### Higher-Dose of Cadmium Induces Anoxia and Cessation of Tissue Perfusion in RVLM

**FIGURE 3:**
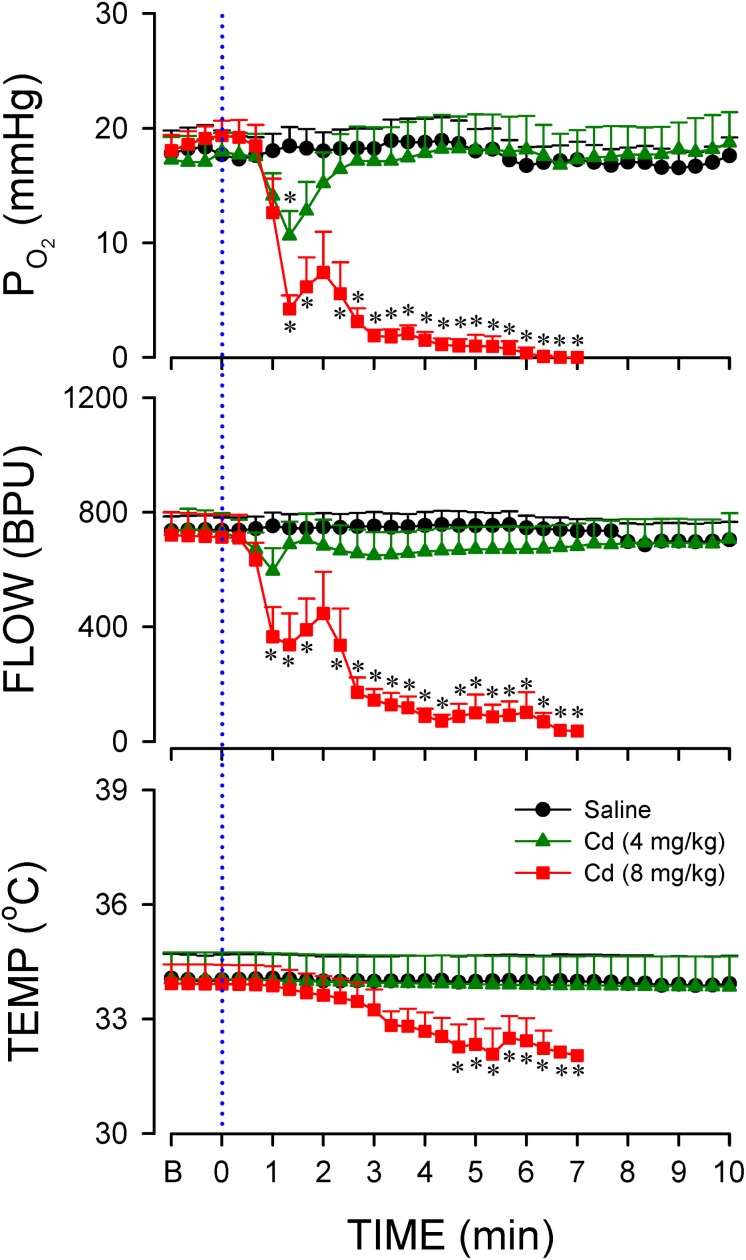
Temporal changes in tissue oxygen tension, microvascular perfusion or temperature in rostral ventrolateral medulla (RVLM) of rats that received intravenous administration of cadmium (Cd; 4 or 8 mg/kg) or saline. Values are mean ± SEM, *n* = 5–7 animals per experimental group. ^*^P < 0.05 vs. saline group at corresponding time points in the *post hoc* Scheffé multiple-range test. Blue dotted line denotes time of cadmium injection. B denotes baseline values.

Previous study (Chang et al., 2009) demonstrated that anoxia and cessation of tissue perfusion in RVLM precede sustained cessation of BLF power and baroreflex dysfunction seen during experimental brain stem death. Intriguingly, our third series of experiments (Figure 3) showed that animals that succumbed to the higher-dose of cadmium (8 mg/kg, iv) also manifested an abrupt anoxia and cessation of local microvascular perfusion in RVLM, followed by a drop in tissue temperature. We also noted that the decrease in local blood flow and tissue oxygen level in RVLM (Figure 3) took place before the decline in BLF power (Figure 2). On the other hand, administration of the lower-dose of cadmium (4 mg/kg, iv) induced only a transient hypoxia that paralleled the MAP response, alongside insignificant changes in local microvascular perfusion or tissue temperature. Administration of saline induced insignificant changes in tissue oxygen level, local blood flow or tissue temperature in RVLM.

### Higher-Dose of Cadmium Induces Necrosis and Lower-Dose of Cadmium Induces Apoptosis in RVLM

**FIGURE 4:**
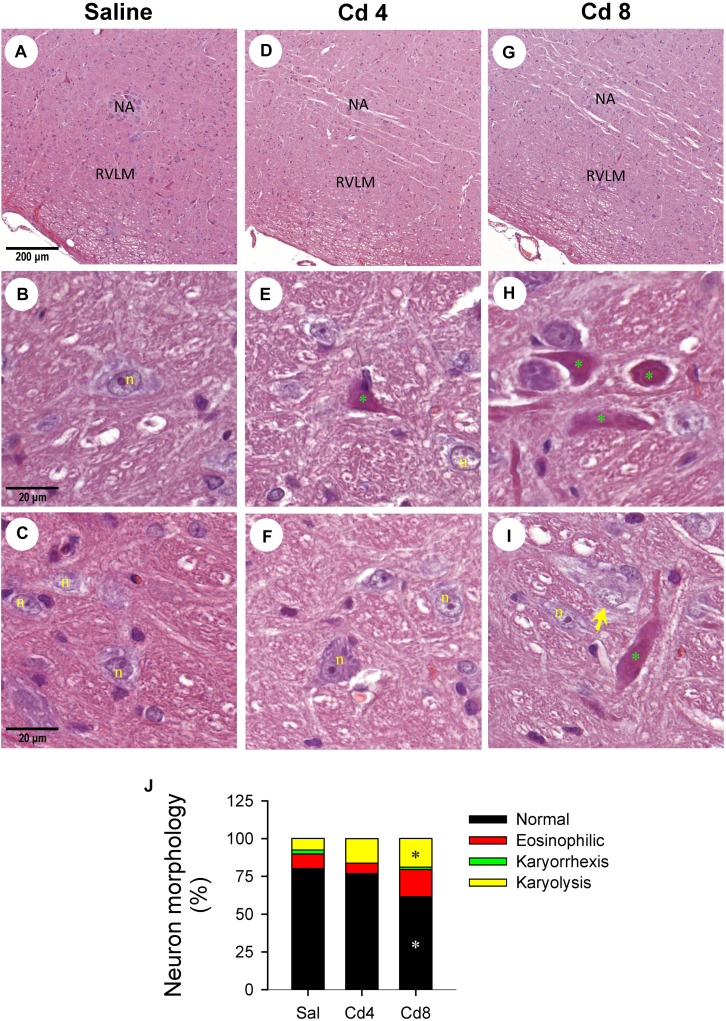
Representative photomicrographs of RVLM stained by hematoxylin and eosin showing nuclear karyolysis and eosinophilic neurons in rats that received saline **(A–C)**, 10 min after cadmium (Cd; 4 mg/kg, iv) **(D–F)**, or died from Cd (8 mg/kg, iv) **(G–I)**. **(J)** Stacked bar plots showing the average distribution of neurons in RVLM (in percentage) based on morphology from hematoxylin and eosin staining. These results are typical of 3–4 animals from each experimental group. ^*^P < 0.05 vs. saline (sal) group. In **(A–I)**, NA, nucleus ambiguus; n, nucleus; green symbol (^*^), eosinophilic neuron; yellow arrow, neuron exhibiting karyolysis.

**FIGURE 5:**
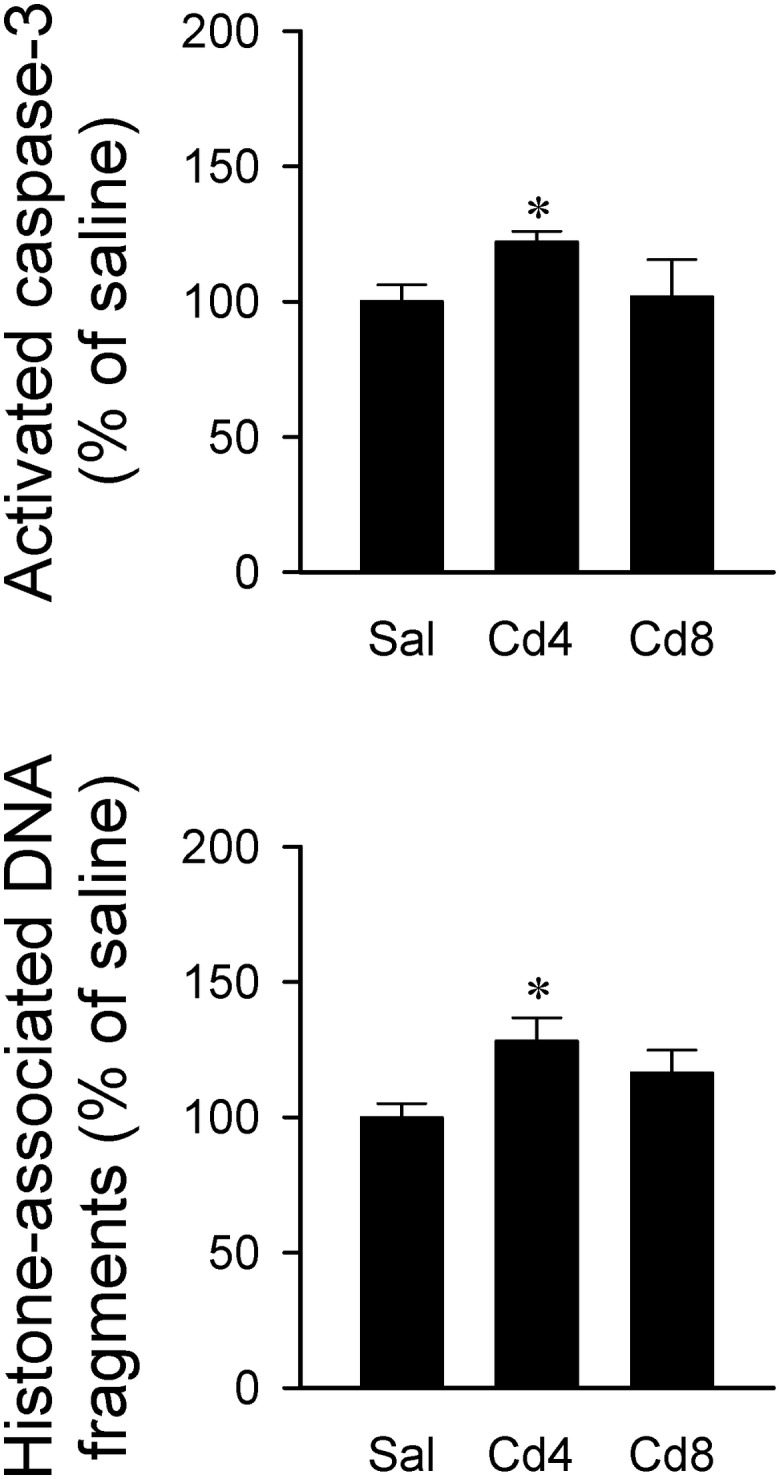
Changes in activated caspase-3 level or histone-associated DNA fragments in cytoplasm against saline-control in tissues collected from RVLM of rats that received saline, 10 min after cadmium (Cd; 4 mg/kg, iv), or died from Cd (8 mg/kg, iv). Values are mean ± SEM, *n* = 4–5 animals per experimental group. ^*^P < 0.05 vs. saline (sal) group in the *post hoc* Tukey test.

**FIGURE 6:**
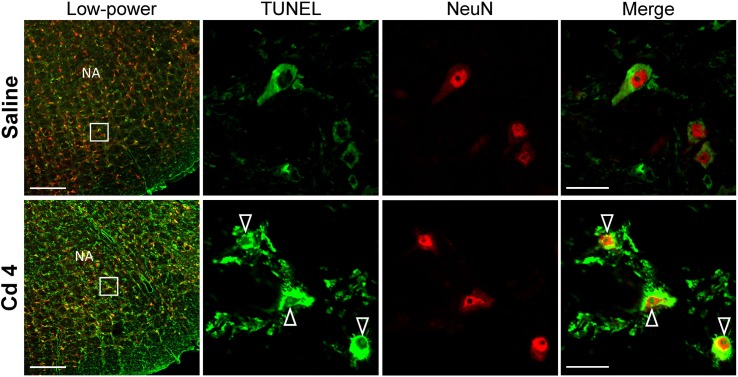
Illustrative examples of *in situ* detection of apoptosis in RVLM neurons using TUNEL assay (green fluorescence) and staining for the neuronal marker, NeuN (red fluorescence) in rats 10 min after saline or cadmium (Cd; 4 mg/kg, iv). White arrows denote TUNEL-positive staining in the nucleus of cells that additionally exhibit NeuN-immunoreactivity. Box in low-power view of the medulla oblongata indicated the location of TUNEL-positive neurons. Scale bar, 20 μm in low-power view or 200 μm in high-power view. NA, nucleus ambiguus.

Our hematoxylin and eosin staining results revealed that animals that received the higher-dose of cadmium (Figures 4G,I) showed necrotic appearing neurons in RVLM, characterized by eosinophilicity (shrunken neurons with loss of normal cytoplasmic and nuclear details, and darkly stained red eosinophilic cytoplasm), karyolysis (nuclear fading), and karyorrhexis (nuclear fragmentation). Quantitative analysis (Figure 4J) revealed that 80.0 ± 2.6% of neurons in RVLM of saline-control rats exhibited clear hematoxylin-labeled nucleolus, intact nuclear membrane, nucleus and cytoplasm (Figures 4A–C), as opposed to 76.7 ± 5.8% in the lower-dose cadmium group (Figures 4D–F), and 61.6 ± 8.6% (*P* < 0.05 vs. saline) in the higher-dose cadmium group (Figures 4G–I). In addition, in animals that received the higher dose of cadmium (8 mg/kg, iv), the number of RVLM neurons that manifested eosinophilicity (17.8 ± 4.7% vs. 9.6 ± 2.5%; *P* < 0.05) and karyolysis (18.9 ± 3.6% vs. 7.6 ± 2.2%; *P* < 0.05) was significantly higher than that in the saline-control group. In contrast, results from activated caspase-3 and histone-associated DNA fragments, two experimental indices for apoptosis, showed that the level of apoptotic cell death in RVLM from the lower-dose cadmium group was significantly augmented (Figure 5). Our results from TUNEL assay (Figure 6) further showed that the increase of apoptotic cell death after administration of cadmium took place in RVLM neurons. However, apoptosis was absent in RVLM of rats that received saline or the higher-dose cadmium.

### Bioenergetic Failure Underpins Higher-Dose of Cadmium-Induced Necrotic Cell Death in RVLM

**FIGURE 7:**
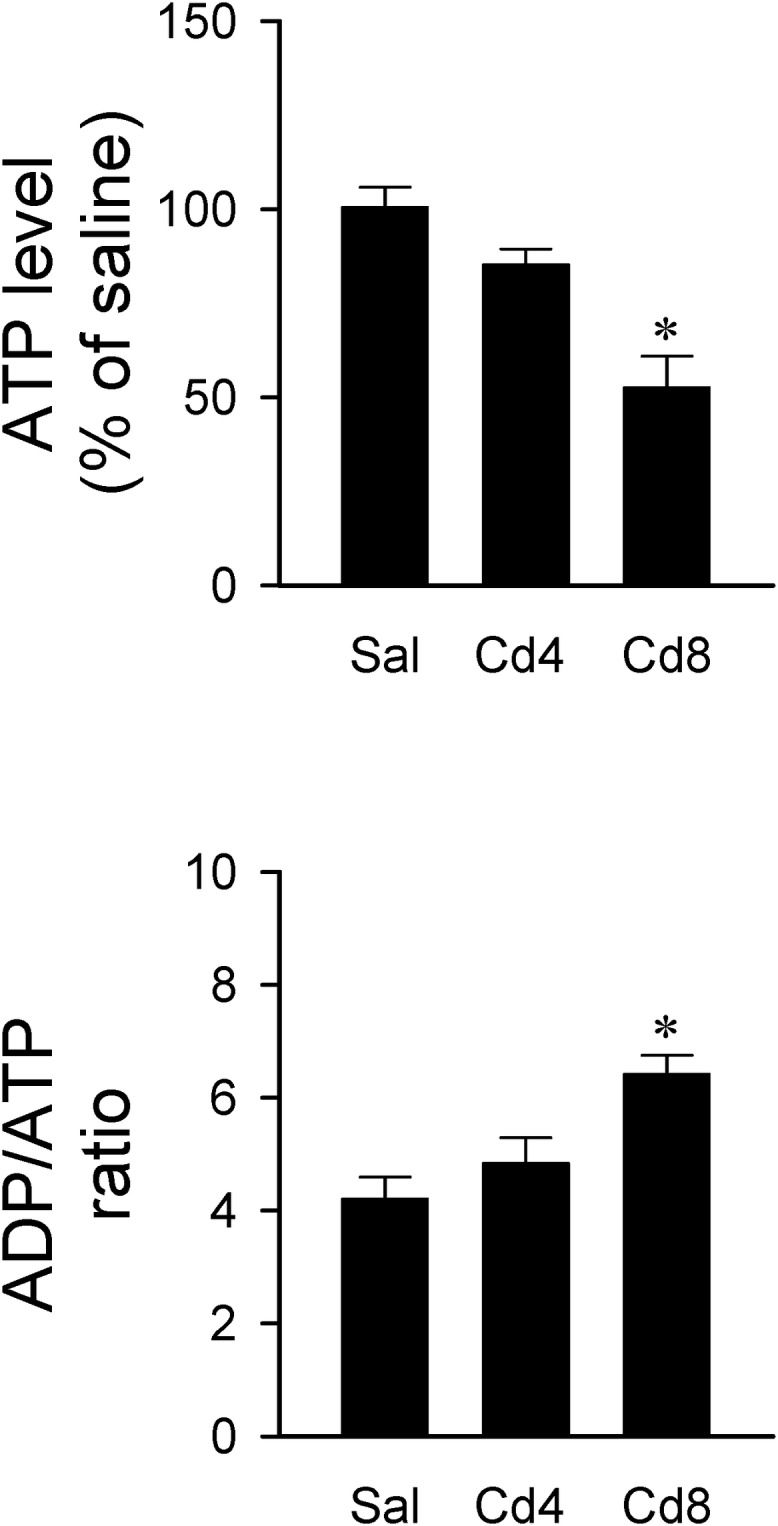
Changes in ATP level against saline-control or ADP/ATP ratio in tissues collected from RVLM of rats that received saline, 10 min after cadmium (Cd; 4 mg/kg, iv), or died from Cd (8 mg/kg, iv). Values are mean ± SEM, *n* = 4–5 animals per experimental group. ^*^P < 0.05 vs. saline (sal) group in the *post hoc* Tukey test.

Since ATP is the primary source of metabolic energy in brain, neurons tend to undergo necrosis in response to hypoxic or anoxic stress (Nagańska and Matyja, 2001). In this series of experiments, we found that rats that received the higher-dose of cadmium (8 mg/kg, iv) exhibited a drastic reduction in total ATP level and an increase in ADP/ATP ratio in RVLM (Figure 7). ADP level in RVLM also increased in the high-dose of cadmium group (data not show). However, there was only a slight reduction in total ATP level and no significant alteration in ADP/ATP ratio in RVLM in the lower-dose of cadmium (4 mg/kg, iv) group.

### Cadmium Induces Decrease of Mitochondrial Membrane Potential in RVLM

**FIGURE 8:**
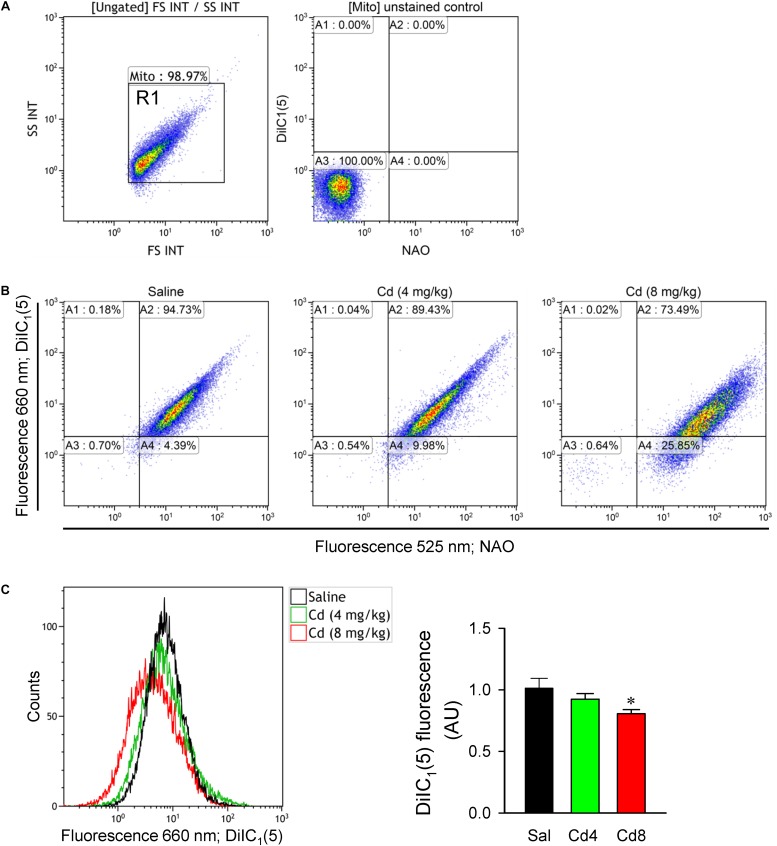
Estimation of mitochondrial membrane potential from freshly isolated mitochondria of RVLM tissue. **(A)** Mitochondria were gated based on light scattering properties in the FS and SS modes by 20,000 events for each sample within the gate R1. Unstained control sample shown fluorescence at 525 and 660 nm for the events within R1. **(B)** Representative dot plots from a double-staining using 100 nM NAO (mitochondrial marker) and 10 nM DiIC_1_(5) (mitochondrial membrane potential indicator). **(C)** Representative histogram or quantification of mean intensity of fluorescence at DiIC_1_(5) (660 nm) from mitochondria in rats that received saline, 10 min after cadmium (Cd; 4 mg/kg, iv), or died from Cd (8 mg/kg, iv). Values are mean ± SEM, *n* = 5–6 animals per experimental group. ^*^P < 0.05 vs. saline (sal) group in the *post hoc* Tukey test.

We further evaluated mitochondrial function in RVLM by measuring changes in mitochondrial membrane potential using flow cytometry. Double staining of mitochondria with NAO (mitochondrial marker) and DiIC_1_(5) (mitochondrial membrane potential indicator) was performed to measure membrane potential only in NAO-stained particles (Figure 8B). In all samples, mitochondria were selected from background based on light-scattering properties in the SS and FS modes (R1; Figure 8A). We found that mitochondrial membrane potential measured by the mean DiIC_1_(5) fluorescence intensity (Figure 8C) was significantly reduced in the higher-dose of cadmium groups. However, there was only a slight and no significant reduction in mitochondrial membrane potential in the lower-dose of cadmium group.

### Lower-Dose of Cadmium Induces Neuroinflammation in RVLM

**FIGURE 9:**
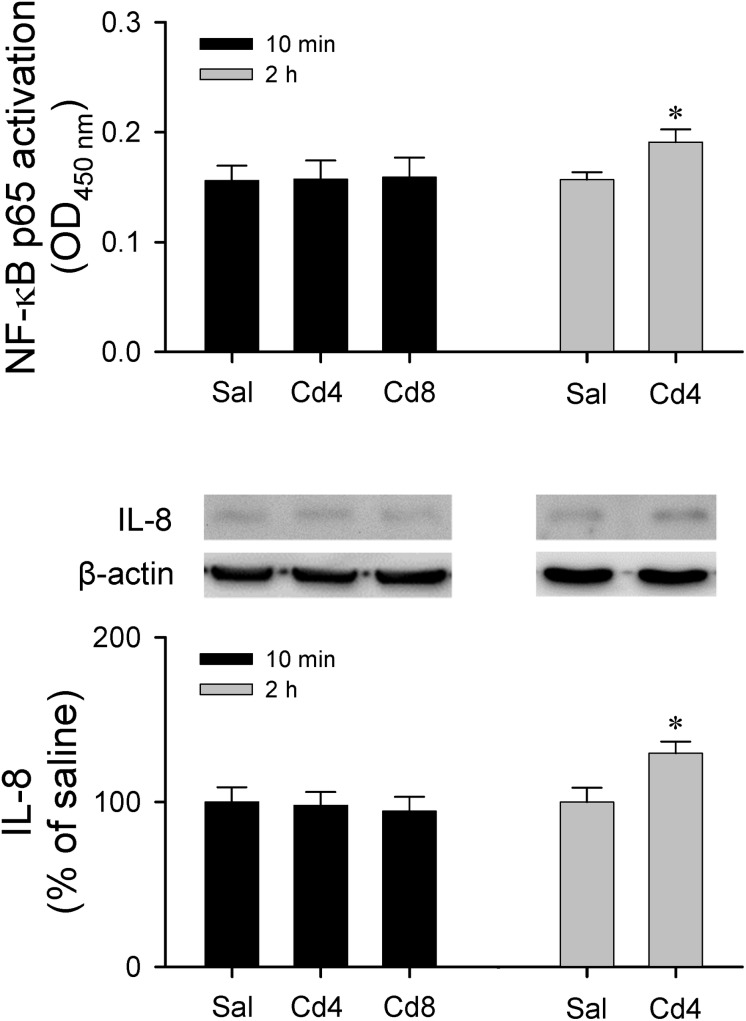
Measurement of transcriptional activity of NF-κB p65 in the RVLM or western blot analysis of expression of IL-8 relative to β-actin in tissues collected from RVLM of rats that received saline, 10 min after cadmium (Cd; 4 mg/kg, iv), or died from Cd (8 mg/kg, iv). Values are mean ± SEM, *n* = 3–4 animals per experimental group. ^*^P < 0.05 vs. saline (sal) group in the *post hoc* Tukey test.

Results from transcriptional activity and western blot analysis (Figure 9) showed that there was no significant alteration in NF-κB activation and IL-8 protein level in RVLM 10 min after lower- or higher-dose of cadmium administration. However, animals that received the lower-dose of cadmium showed a significant increase in NF-κB activation and IL-8 protein expression in RVLM 2 h after cadmium administration.

### Lack of Changes in Blood pH, Sodium and Potassium, or Plasma Level of Thrombomodulin After Acute Administration of Cadmium

Analysis of blood samples (Table 1) collected 5 min after the administration of the lower- or higher-dose of cadmium showed no significant changes in blood pH, sodium and potassium or plasma level of thrombomodulin.

**TABLE 1:** Analyses of blood samples collected from rats 5 min after intravenous administration of cadmium (Cd; 4 or 8 mg/kg) or saline.

	**Saline**	**Cd (4 mg/kg)**	**Cd (8 mg/kg)**
pH	7.37±0.03	7.35±0.01	7.40±0.01
Sodium (mmol/L)	142.3±0.3	140.3±0.8	142.3±0.8
Potassium (mmol/L)	4.00±0.05	4.23±0.28	4.10±0.20
Thrombomodulin (pg/mL)	182±16	193±10	185±22

*Values are mean ± SEM, *n* = 3 animals per experimental group. No significance among all groups (*P* > 0.05) in ANOVA analysis.*

## Discussion

A majority of the reported mechanisms for cadmium-induced cardiovascular toxicity is based on either long-term exposure to cadmium in animals (Puri, 1999; Yoopan et al., 2008; Ozturk et al., 2009), or from *ex vivo* (Kisling et al., 1993; Wakabayashi et al., 1995) or *in vitro* (Majumder et al., 2008; Messner and Bernhard, 2010; Messner et al., 2016) studies. Much less studies address the acute circulatory actions of cadmium. The present study fills this void by providing physiological and biochemical observations which revealed that acute exposure to cadmium resulted in drastically different dose-dependent circulatory fates that entail differential engagement of central cardiovascular regulatory mechanisms.

Previous studies (Kuo et al., 1997b; Chan et al., 2005) demonstrated that disappearance of the baroreflex-medicated sympathetic vasomotor tone precedes brain stem death in comatose patients who succumbed to severe brain injury (Yien et al., 1997), systemic inflammatory response syndrome (Kuo et al., 1997b), or organophosphate poisoning (Yen et al., 2000). The present results showed that a time-window represented experimentally by the power density of the BLF component also exists between the administration of cadmium and death, which was inversely related to the doses used. On administration of the higher-dose of cadmium, our results showed that blood pressure dropped quickly in the first min after injection, BLF sustained for 3 min, and the animal finally died after 10 min. However, under a lower-dose of cadmium, the MAP, HR, and BLF were maintained to reflect high survival rate.

The brain uses 20% of available oxygen for normal function, making tight regulation of blood flow and oxygen delivery critical for survival (Widmaier et al., 2006; Bélanger et al., 2011). Flow autoregulation is the mechanism by which organs and tissues alter their own arteriolar resistances, thereby self-regulating their blood flow (Widmaier et al., 2006). Our results from CBF measurements also showed dose-dependent changes in cerebral autoregulation that paralleled survival after cadmium administration. Intriguingly, the increased in CBF after administration of the lower-dose cadmium was reflected in sustained tissue oxygen level and local blood flow in RVLM, which peaked before maximal BLF response occurred. On the other hand, the progressive decrease in CBF was echoed by a gradual decline in tissue oxygen, local blood flow, tissue temperature and BLF power, leading to high mortality rate after administration of the higher-dose of cadmium.

The brain is highly dependent on energy for normal activity, and mitochondria are the major source of ATP production. By revealing similar patterns of reduction in ATP level and mitochondrial membrane potential, our results suggest that cadmium induces mitochondrial dysfunction that leads to decrease of ATP production in RVLM. Previous studies (Yen et al., 2004; Chang et al., 2009) demonstrated that mitochondrial dysfunction in RVLM is a major culprit of baroreflex dysregulation. It follows that mitochondrial dysfunction in brain stem sites involved in cardiovascular regulation remains to be a potential target for cadmium toxicity.

*In vitro* study showed that whereas lower concentrations of cadmium (<1 μM) induce apoptosis, higher concentrations of cadmium (>1 μM) induce necrotic cell death (López et al., 2003). Furthermore, intracellular ATP levels have been implicated as a determinant for apoptosis or necrosis (Tsujimoto, 1997; Tatsumi et al., 2003). It is therefore of interest that our histological staining results revealed that the higher-dose of cadmium induced necrotic cell death in RVLM neurons, along with significant decrease in ATP level. In contrast, with only slight reduction of ATP level, rats that received the lower-dose of cadmium exhibited an increase in activated caspase-3 and histone-associated DNA fragments in RVLM, and results from TUNEL assay indicated the occurrence of apoptotic cell death in RVLM neurons. However, we recognize that both neurons and astrocytes can release ATP during basal metabolic activity and during neuronal activation (Shetty et al., 2012). It is therefore possible for apoptotic cell death to take place in neurons and astrocytes in RVLM.

Necrotic cell death occurs in response to many kinds of insults (e.g., trauma, infarction, and toxins, etc.) and therefore is typically the result of a pathological process (Rock and Kono, 2008). Morphologically, it is associated with cell swelling and/or the rapid loss of membrane integrity and an uncontrolled release of products of cell death into the extracellular space (Proskuryakov et al., 2003). In contrast to apoptosis, necrosis is almost always detrimental and can be fatal. Chang et al. (2009) demonstrated that bioenergetic failure leading to necrotic cell death accounts for the loss of functional integrity in RVLM, leading to failure to resume spontaneous circulation after cardiac arrest in experimental brain stem death. They also used tissue oxygen level as a determinant of functional integrity of RVLM. Our results showed that the drop of local blood flow or tissue oxygen level in RVLM occurred earlier than the cardiovascular responses and before animals succumbed to the higher-dose of cadmium. When combined with our ATP and histological staining results, these observations support that bioenergetic failure, leading to necrotic cell death, accounts for the loss of functional integrity in RVLM, and death in animal. In the present study, acute intoxication was induced by intravenous administration of cadmium. It is therefore possible that cadmium may also affects the afferent component of baroreflex such as baroreceptors located in aortic arch and carotid sinus or the nucleus tractus solitarii where baroreceptor afferents terminate in the brain stem (Dampney, 1994; Spyer, 1994).

Cadmium is a potent inhibitor of voltage-dependent calcium channel (Yang et al., 1993). Previous study (Yuan et al., 2013) reported that cadmium elicits neurotoxicity through disruption of intracellular free calcium homeostasis, leading to apoptosis via calcium-mitochondria signaling. Our flow cytometry results also showed a reduction of mitochondrial membrane potential, suggesting mitochondrial dysfunction in RVLM. It is therefore conceivable that cadmium may induce cardiovascular impairment via calcium-mitochondria signaling in RVLM neurons. We also noted that voltage-dependent calcium channels expressed in chemoreceptor cells of rabbit carotid body have been shown to be affected by cadmium (Rocher et al., 2005). Our results from blood sample analysis showed that the blood pH in animals was not significantly changed at 5 min after intravenous administration of lower- or higher-dose of cadmium. The contribution of chemoreflex to our observations is therefore deemed minimal.

Cadmium can pass the BBB and accumulate in the CNS via increasing permeability of BBB (Shukla et al., 1996) to induced neurotoxicity (Wang and Du, 2013). It has also been reported that cadmium stimulates intercellular adhesion molecule-1 (ICAM-1) (Jeong et al., 2004) expression via NF-κB activation in cerebrovascular endothelial cells, leading to BBB injury. Recent studies showed that cadmium induces neuroinflammation via activation of astrocytes or microglia (Ashok et al., 2015; Favorito et al., 2017). Phuagkhaopong et al. (2017) also reported that the cadmium-induced interleukin 6 (IL-6) and interleukin 8 (IL-8) expression and release from astrocytes are mediated by NF-κB at 6 and 24 h post-exposure of cadmium. Our results showed that there was no significant alteration in NF-κB activation and IL-8 protein level in RVLM 10 min after lower- or higher-dose of cadmium administration. However, animals that received the lower-dose of cadmium showed a significant increase in NF-κB activation and IL-8 protein expression in RVLM after 2 h of cadmium administration. The causal relationship between neuroinflammation and BBB injury in RVLM and impairment of cardiovascular regulation during cadmium-induced sub-lethal cardiovascular toxicity remains to be established in the future.

Epidemiological studies from humans (Nakagawa and Nishijo, 1996; Tellez-Plaza et al., 2008) and studies in animals (Puri, 1999; Yoopan et al., 2008) have revealed that chronic exposure to cadmium causes hypertension. Its mechanism has been reported to include endothelial dysfunction (Lukkhananan et al., 2015), suppression of eNOS expression in endothelial cells (Majumder et al., 2008) or in blood vessels (Yoopan et al., 2008), and vascular relaxation (Yoopan et al., 2008). Of note is that our results showed no significant changes in plasma thrombomodulin, a marker for endothelial dysfunction (John et al., 1999), at 5 min after administration of the lower- or higher-dose of cadmium. The contribution of endothelial dysfunction to our results on acute cardiovascular effects of cadmium is therefore deemed minimal.

The kidney is another organ of injury after cadmium exposure (Winiarska-Mieczan and Kwiecień, 2016); hyperkalemia associated with renal injury was reported in rats 5 h after intravenous administration of cadmium (Dote et al., 2007). In addition, previous study in human renal glomerular endothelial cells indicated that NF-κB signaling plays an essential role in maintaining the survival of low-dose cadmium exposed (Zhang H. et al., 2016). However, our results showed no significant alterations in NF-κB activation (Figure 9) or blood electrolytes (Table 1) 5–10 min after cadmium administration. As such, it is unlikely that our observed dose-dependent acute cardiovascular effects induced by cadmium are related to renal injury.

We are cognizant that there are at least two limitations in the present study. First, it is difficult to carry out real-time detection of cadmium concentrations in the RVLM. Second, cadmium has been administered in different routes and doses for chronic or acute exposure studies in humans, animals or *in vitro* preparations. The doses of cadmium used in the present study were adopted from the report by Puri (1999) and selected from preliminary experiments as representative doses that elicited differential effects on our experimental parameters within 10 min after administration.

In conclusion, the present study provided physiological and biochemical evidence that support the notion that cadmium elicits drastically different circulatory fates that are dose-dependent and entail differential engagement of central cardiovascular regulatory mechanisms. We demonstrated that a lower-dose of cadmium elicited low mortality, transient decrease in MAP, and hypoxia and apoptosis in RVLM neurons that reflect sustained cerebral autoregulation. In contrast, a higher-dose of cadmium induced acute cardiovascular collapse by the loss of functionality in RVLM because of anoxia, diminished tissue perfusion, mitochondrial dysfunction and bioenergetic failure that echo loss of cerebral autoregulation, leading to necrosis that further exacerbated mortality.

## Ethics Statement

All experimental procedures carried out in this study were approved by the Institutional Animal Care and Use Committee of the Kaohsiung Chang Gung Memorial Hospital (Approval Number: 2017091402), and were in accordance with the guidelines for animal care and use set forth by that committee.

## Author Contributions

S-MC collected, analyzed, and interpreted the data, and drafted the manuscript. SP, CF, JW, and Y-HH collected, analyzed, and interpreted the data. PV and H-HL conceived and designed the study and critically revised the intellectual content of the manuscript. C-YT conceived and designed the study, critically revised the intellectual content of the manuscript, supervised the overall study, and drafted the manuscript. All authors revised and approved the final version of the manuscript.

## Conflict of Interest Statement

The authors declare that the research was conducted in the absence of any commercial or financial relationships that could be construed as a potential conflict of interest.
